# Reliable Dimerization Energies for Modeling of Supramolecular Junctions

**DOI:** 10.3390/ijms25010602

**Published:** 2024-01-02

**Authors:** Jiří Czernek, Jiří Brus

**Affiliations:** Institute of Macromolecular Chemistry, Czech Academy of Sciences, Heyrovsky Square 2, 16200 Prague, Czech Republic; brus@imc.cas.cz

**Keywords:** noncovalent interactions, supramolecular junctions, interaction energy, DFT, CCSD(T)

## Abstract

Accurate estimates of intermolecular interaction energy, Δ*E*, are crucial for modeling the properties of organic electronic materials and many other systems. For a diverse set of 50 dimers comprising up to 50 atoms (Set50-50, with 7 of its members being models of single-stacking junctions), benchmark Δ*E* data were compiled. They were obtained by the focal-point strategy, which involves computations using the canonical variant of the coupled cluster theory with singles, doubles, and perturbative triples [CCSD(T)] performed while applying a large basis set, along with extrapolations of the respective energy components to the complete basis set (CBS) limit. The resulting Δ*E* data were used to gauge the performance for the Set50-50 of several density-functional theory (DFT)-based approaches, and of one of the localized variants of the CCSD(T) method. This evaluation revealed that (1) the proposed “silver standard” approach, which employs the localized CCSD(T) method and CBS extrapolations, can be expected to provide accuracy better than two kJ/mol for absolute values of Δ*E*, and (2) from among the DFT techniques, computationally by far the cheapest approach (termed “ωB97X-3c/vDZP” by its authors) performed remarkably well. These findings are directly applicable in cost-effective yet reliable searches of the potential energy surfaces of noncovalent complexes.

## 1. Introduction

Intermolecular noncovalent interactions are of importance in current investigations in biology (see the book [1]), chemistry (see the review by Stoddard et al. [2]), physics (see reference [3] and the works cited therein), and materials research [4,5,6]. In the area of materials science, certain types of noncovalent interactions are crucial for the design of novel electronic materials [7,8,9,10,11,12]. Specifically, supramolecular junctions containing π-conjugated molecules have frequently been studied (see the review [13]). The key parameter in many of the aforementioned investigations is an estimate, either experimental or theoretical, of the intermolecular binding energy [14]. Here, this parameter is referred to simply as the interaction energy, Δ*E*, expressed in units of kJ/mol. From among the computational approaches to an assessment of the Δ*E* value, the coupled cluster theory with singles, doubles, and perturbative triples [CCSD(T)] is particularly important [15]. This is because the CCSD(T) energies extrapolated to the complete basis set (CBS) limit can be used to obtain benchmark Δ*E* values, which are considered to be fully reliable for systems of any composition and geometry [16,17,18,19,20,21,22] and may serve for gauging the quality of results provided by more approximative methods (see reference [23] for the most recent discussion of various quality standards for the CCSD(T)-based interaction energies). Thus, a number of databases of the canonical CCSD(T)/CBS Δ*E* data are available (see references [21,22,24], which also cite older databases). However, all of them are (i) limited in terms of the size of the investigated complexes (to 30 atoms [24]) and (ii) do not include systems related to the aforementioned research of supramolecular junctions (see the most recent investigation [25]). In order to fill these gaps, the canonical CCSD(T)/CBS Δ*E* values of the “golden standard” quality are compiled here, which were obtained for high-quality geometries of a diverse set of 50 dimers comprising up to 50 atoms. This set is, thus, termed Set50-50, and it also contains models of single-stacking junctions (see Section 2 for a description of three distinct groups of intermolecular complexes included in the Set50-50). By employing the benchmark data, the performance of several computational methods was evaluated and thoroughly discussed for the Set50-50. One of the applied methods belongs to the group of reduced-scaling variants of the CCSD(T). While these variants were most recently discussed in references [23,26], it should be pointed out that the local natural orbital CCSD(T) technique (see reference [27] and related works cited therein) was employed for an estimation of the CCSD(T)/CBS Δ*E* of systems with over 100 atoms [28]. In the present work, the domain-based local pair natural orbital (DLPNO) scheme [29,30,31] was used together with an iterative treatment of triple excitations within the CCSD(T) method [32] to establish a level of absolute accuracy of the resulting scheme, abbreviated as DLPNO-CCSD(T). Other computational methods considered here are part of the large family of dispersion-corrected density-functional theory (DC DFT) approaches [33,34,35]. Specifically, the ωB97X-3c/vDZP strategy, most recently proposed by Grimme et al. (see reference [34] and Section 4), was tested, and its results were put into perspective by comparing them to those obtained from two well-established but computationally much more demanding DC DFT methods: B3LYP-D3 and B2PLYP-D3(BJ); these were applied together with an ample basis set (see Section 4 for the specifications). Moreover, the symmetry-adapted perturbation theory (SAPT) [36] was combined with the DFT description of monomers [37] in order to describe the physical origin of the intermolecular binding of the Set50-50 dimers. Thus, the main aims of the current paper are (i) to present important reference data of the Set50-50 and (ii) to compare the performance of various computational methods when applied to this set. Importantly, the proposed focal-point approach that combines results of the Hartree–Fock (HF), the DLPNO-based second-order Møller–Plesset (MP2) [38], and the DLPNO-CCSD(T) calculations (details are provided in Section 4), delivers the Δ*E* data with an absolute accuracy of better than two kJ/mol also for the biggest complexes. Furthermore, the computationally very cheap ωB97X-3c/vDZP method performs comparably to the “traditional” DC DFT approaches and is, thus, suitable for a cost-effective screening of the potential energy surfaces. These findings are expected to be useful in the modeling of supramolecular junctions and other complex systems [39].

## 2. Results

### 2.1. Smaller Dimers of Set50-50

For all systems of the Set50-50, the underlying absolute energies for an estimation of the canonical CCSD(T)-, DLPNO-CCSD(T)-, and DFT-based interaction energies are provided in the Supplementary Materials files “canonical.xlsx”, “DLPNO.xlsx”, and “DFT.xlsx”, respectively, and the geometries are available in the respective sheets of the “canonical.xlsx” file. At this point, it should be stressed that the benchmark CCSD(T)/CBS results were obtained while applying the large aug-cc-pVTZ (augmented correlation-consistent polarized valence triple-zeta) basis set [40,41] in the canonical CCSD(T) calculations (see Equation (1) in Section 4). Table 1 shows the dimerization energy values predicted by the aforementioned theoretical methods for complexes whose size does not exceed 32 atoms, while Table S1 specifies the sources of the geometry of these complexes. These systems were included mainly for checking the current computational methodology, as their canonical CCSD(T)/CBS Δ*E* values were estimated in previous investigations (see references [42,43,44] and the works cited therein, along with the footnotes to Table 1). Importantly, the Δ*E* data span a large interval from ca. −41 to ca. −6 kJ/mol. On the basis of the SAPT-DFT/CBS calculations, which are detailed in Section 4, the smaller dimers are divided into three groups. In Table 1, values of the dispersion-to-polarization ratio [45] are shown that are used to classify the investigated complexes as electrostatic-dominated, of mixed electrostatic-dispersion character, and dispersion-bound. It should be noted that there is a satisfactory agreement between the SAPT-DFT/CBS total interaction energies of this set of dimers, $\left\{y\right\}$, and the corresponding benchmark values, $\left\{x\right\}$, as follows from parameters of their linear relationship: $\left\{y\right\}=0.981\times \left\{x\right\}-0.285$ kJ/mol with an adjusted $R^{2}\;$ = 0.993 and standard deviation of 0.797 kJ/mol. The maximum residual of this regression is found for anisole⋯CO_2_ adduct and amounts to 1.73 kJ/mol, which is 10.9% of the corresponding benchmark value of ca. −15.9 kJ/mol. It should also be noted that an uncertainty in the benchmark data themselves is very small, amounting to about a quarter of kJ/mol. This can be inferred from a comparison of the present CCSD(T)/CBS results for several complexes coming from the S66x8 set [45] to values that were most recently reported in references [23,24] (see Table 1). Thus, for each subset of the dimers, differences between the canonical CCSD(T)/CBS Δ*E* data and their counterparts obtained by the DLPNO-CCSD(T)/CBS and three types of DC DFT calculations can be reliably evaluated. The maximum absolute values of these differences and their standard deviations are shown in Table 2 to enable a head-to-head comparison between respective methods. It is stressed that for any interaction type and applied computational method, there is no outlier that would exhibit a vastly different quality of predicted data. Expectedly, the DLPNO-CCSD(T)/CBS technique performs the best. It provides a result differing from the benchmark value by more than 1.0 kJ/mol only in 1 instance out of 23, namely, for the challenging [44] stacked dimer of uracil. It is worth mentioning that the discrepancy of 1.366 kJ/mol found for this system leads to only a small relative error of 3.4% relative to the benchmark value of −40.570 kJ/mol, and that the highest relative error in the case of these DLPNO-CCSD(T) results occurs for the stacked dimer of pyridine and amounts to 4.6%, as follows from an inspection of data in Table 1. The results of the present DLPNO-CCSD(T) computations can, thus, be considered to be of a “silver standard” quality. The B3LYP-D3/def2-QZVPPD approach previously delivered surprisingly accurate dissociation energies [42] and, hence, was tested here. In one case, namely, for the HCl dimer with a benchmark Δ*E* of 7.940 kJ/mol, the relative error of this approach slightly exceeds 10%, as the Δ*E* of 8.824 kJ/mol is predicted. The biggest absolute difference between these two data sets amounts to 1.810 kJ/mol and occurs for the 1-naphtol⋯ammonia complex that features a high Δ*E* value (see Table 1), which leads to a relative error smaller than 5% in this case. Interestingly, an application of the computationally demanding B2PLYP-D3(BJ)/def2-QZVPPD approach, which entails the MP2 calculation, does not lead to significant improvements in predicted results, as compared to those obtained by the B3LYP-D3/def2-QZVPPD method (see Table 2). The highest absolute error of the B2PLYP-D3(BJ)/def2-QZVPPD Δ*E* data with respect to the benchmark values is found in anisole⋯CO_2_ dimer (1.725 kJ/mol), while the relative error exceeds 10% in even three dimers, namely, anisole⋯CO_2_, aniline⋯CH_4,_ and anisole⋯CH_4_ (in this case, it is the highest, amounting to 14.3%). In the context given above, a performance of the computationally very cheap ωB97X-3c/vDZP technique is quite promising. Specifically, results obtained for the subset of dispersion-bound complexes do not substantially differ from those of the other two DFT-based methods (see Table 2). This point was, thus, investigated further by considering a number of configurations of sizeable molecular clusters formed by 9*H*-xanthene and either phenol or toluene, as described in the subsequent section.

### 2.2. Stacked Complexes of 9H-Xanthene

The stacking ability of numerous dimers formed between heterocycles was computationally studied in reference [46]. In particular, the B97-D/def2-TZVPP method (that is, the DC DFT approach combining the B97 exchange-correlation functional [47] with the dispersion correction from reference [48] and the basis set from reference [49]) was employed to locate relevant minima of the potential-energy surface (PES). For 9*H*-xanthene⋯phenol and 9*H*-xanthene⋯toluene dimers, the number of such minima was eight and eleven, respectively. Their geometries were employed here for estimation of the Δ*E* of these larger (containing either 39 or 40 atoms), complicated clusters. Computed interaction energies are listed in Table 3, together with values of the dispersion-to-polarization ratio, as obtained from the SAPT-DFT/CBS calculations. All 18 structures can be categorized as bound by van der Waals dispersion [50]. The canonical CCSD(T)/CBS benchmark data span an interval from ca. –41.8 to –28.7 kJ/mol (see Table 3). They have a mean, median, and standard deviation value equal to ca. –33.8, –34.1 and 4.0 kJ/mol, respectively, and are quite uniformly distributed, with an average difference between sorted values amounting to ca. 0.7 kJ/mol. Hence, they are suitable for an evaluation of the relative interaction energies together with the absolute accuracy of predicted Δ*E*. Table 4 summarizes the performance of the DLPNO-CCSD(T) and DFT-based methods in reproducing these benchmark data. Interestingly, this task is quite challenging, even for the “silver standard” DLPNO-CCSD(T)/CBS calculations, as can be immediately seen from an intercept of ca. –1.0 kJ/mol of the linear regression model in the situation when a slope is practically equal to unity (see Table 4). Furthermore, an inspection of the results from Table 3 reveals that only 7 out of the total of 18 data points are correctly ordered. From an analysis of the predicted differences it follows that the DLPNO-CCSD(T)/CBS relative interaction energies are accurate to about one half of kJ/mol with respect to the canonical CCSD(T)/CBS values. Nevertheless, the absolute accuracy of the DLPNO-CCSD(T)/CBS Δ*E* data is fairly high. In particular, the maximum error is ca. 1.1 kJ/mol, which occurs for the configuration #1 of 9*H*-xanthene⋯toluene and amounts to roughly 3% of the Δ*E* of this dimer (see Table 3). It should be noted that the DLPNO-CCSD(T)/CBS Δ*E* are higher in absolute value than their benchmark counterparts, with an average offset of ca. 0.6 kJ/mol, but with one exception, which is the configuration #8 of 9*H*-xanthene⋯phenol (see Table 3). Remarkably, the same pattern of differences is exhibited by the ωB97X-3c/vDZP results, albeit with a higher offset of ca. 0.9 kJ/mol, and the number of data points correctly ordered by this approach is eight. The maximum error of the ωB97X-3c/vDZP calculations occurs for the configuration #4 of 9*H*-xanthene⋯phenol and equals ca. 1.7 kJ/mol, which is slightly less than 5% of the related benchmark value. Thus, the ωB97X-3c/vDZP method performs very well for both relative and absolute values of the Δ*E* data considered in this section. The B3LYP-D3/def2-QZVPPD results are also quite good (see Table 3 and Table 4). Specifically, they feature the lowest absolute value of a maximum error from among of the four tested methods, which amounts to ca. 0.9 kJ/mol only. Regarding the B2PLYP-D3(BJ)/def2-QZVPPD calculations, it has to be mentioned that they systematically overestimate the benchmark results by ca. 1.5 kJ/mol on average and by up to 2.2 kJ/mol, and the related linear fit has a large intercept of ca. 2.9 kJ/mol, but the slope is reasonably close to unity (see Table 4). Nevertheless, some even more demanding tests are described in the section that follows.

### 2.3. Complexes with More than 40 Atoms

The eight biggest dimers of the Set50-50 feature the *C_i_* molecular symmetry that enabled an estimation of their canonical CCSD(T)/CBS Δ*E* in the same way as for clusters described in Section 2.1 and Section 2.2. These large complexes are 2-(4-(methylthio)phenyl)thiophene (“S-T1”) dimer (44 atoms) in five orientations, which are described in reference [25]; *n*-heptane dimer (46 atoms) in the configuration of chains of the monoclinic polymorph of polyethylene [51]; and two 4-(4-(methylthio)phenyl)pyridine (“PY-2”) dimers (50 atoms), one stacked and one hydrogen-bonded (H-bonded), which are adopted from reference [11] and pictured in Supporting Materials Figure S1. Results are presented in Table 5, while Table 6 contains key parameters of the linear regression of pertinent data sets. It should be noted that the *n*-heptane dimer has a very high dispersion-to-polarization ratio (see Table 5). Hence, the *n*-heptane dimer can be expected to be a particularly challenging system for the Δ*E* predictions [28] and is considered together with adducts containing 4-methylthiophenyl fragments, which are of importance for single-stacking supramolecular junctions [9,11,52]. An inspection of data in Table 5 and Table 6 clearly shows that only the “silver standard” DLPNO-CCSD(T) Δ*E* values are in a quantitative agreement with their benchmark counterparts. In particular, the maximum absolute difference between these two data sets occurs for the *n*-heptane dimer and amounts to ca. 0.8 kJ/mol, which is only ca. 4% of its Δ*E* value, while this difference for the B3LYP-D3/def2-QZVPPD approach equals ca. 3.0 kJ/mol and is also found the for *n*-heptane dimer, thus leading to a relative difference of ca. 12% (see Table 5). When the B2PLYP-D3(BJ)/def2-QZVPPD and ωB97X-3c/vDZP methods are applied, the maximum absolute difference occurs for the configuration #5 of the ST-1 dimer and is as large as ca. 5.1 and 5.5 kJ/mol, respectively, which means that the relative error of pertinent Δ*E* data amounts to ca. 12% and 16%, respectively. However, all DFT-based methods are qualitatively correct by providing the same ordering of predicted Δ*E* values as the canonical CCSD(T)/CBS approach. This indicates that the ωB97X-3c/vDZP method, whose computational cost is negligible, should be useful for preliminary scans of the PES, even of complicated systems. A demonstration of such an approach to modeling of azulene- and naphthalene-based dimers [10] is presented in the following section.

## 3. Discussion

**Figure 1:**
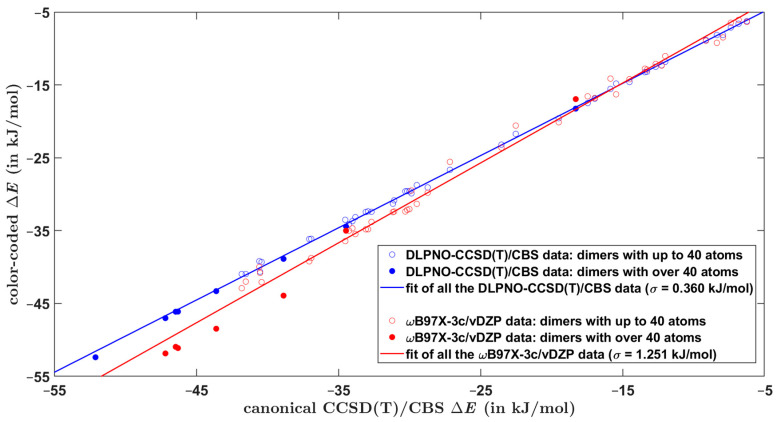
Comparison of computational results for dimers from the Set50-50. The regression line plotted in blue color is $\left\{y\right\}=0.991\times \left\{x\right\}+0.080$ kJ/mol, while the regression line plotted in red color is $\left\{y\right\}=1.097\times \left\{x\right\}+1.714$ kJ/mol.

**Figure 2:**
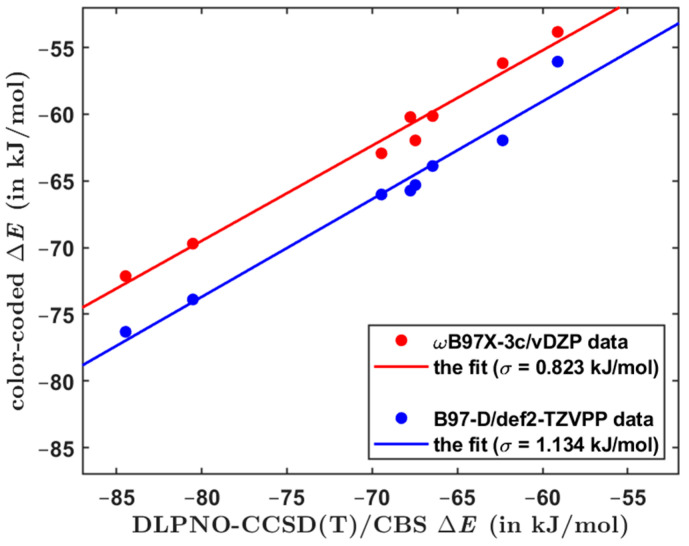
Computational results for azulene- and naphthalene-based dimers that are discussed in the text. The linear regression visualized in red and blue color has the adjusted *R*^2^ value of 0.980 and 0.964, respectively.

The overall performance of computational methods when applied to the Set50-50 dimers is summarized in Table 7 in terms of average and maximum absolute and relative differences with respect to the benchmark Δ*E* values. As anticipated, the focal-point DLPNO-CCSD(T)/CBS computations provide results of a high and uniform quality for all investigated types of dimers. This is particularly important for obtaining reliable estimates of the ∆E of larger models of single-stacking junctions that still could be handled by the DLPNO-CCSD(T)/CBS technique. When applied to structurally similar systems, this approach is accurate to within ca. 0.5 kJ/mol of relative Δ*E* values (see Section 2.2). Regarding the DFT-based methods, results in Table 7 demonstrate their comparable performance. Importantly, despite relatively large errors in underlying absolute values of the dimerization energy, the ωB97X-3c/vDZP approach is quite successful in predictions of differences in the Δ*E* (this is apparent from Figure 1, which plots pertinent data together with the linear regression results). Hence, the ωB97X-3c/vDZP method should be suitable for a high-throughput screening of the PES of supramolecular junctions. This is illustrated by considering a series of π-stacked dimers, which were most recently studied by a combination of theory and experiments in reference [10]. The constituting monomers are denoted as AZ1, AZ2, AZ3, and NA1, as in reference [10] (their chemical names are given in Section 4). For a number of starting orientations of AZ1:AZ1, AZ2:AZ2, AZ3:AZ3, and NA1:NA1 homodimers, the B97-D/def2-TZVPP method was used to locate minima of the PES in the way specified in Section 4. Due to the size of these complexes (either 64 or 72 atoms), the canonical CCSD(T) calculations would not be feasible, and, instead, the DLPNO-CCSD(T)/CBS approach was employed to estimate the reference Δ*E* values. Two minima of each dimer are considered, whose Cartesian coordinates are provided in Supporting Materials “DLPNO_miscellanous.xlsx” file, together with pertinent absolute energies for the DLPNO-CCSD(T)/CBS Δ*E* estimation. For these minima, the interaction energy is also predicted by the three DFT-based methods considered so far and additionally by the B97-D/def2-TZVPP method that was employed in searching the PES. Supporting Materials Table S2 collects all raw Δ*E* values, while Table S3 shows results of their statistical evaluation. The performance of the ωB97X-3c/vDZP and B97-D/def2-TZVPP methods in predicting the dimerization energy is graphically presented in Figure 2 with respect to the DLPNO-CCSD(T)/CBS benchmark data. This figure indicates that the ωB97X-3c/vDZP approach quite accurately reproduces relative differences in the Δ*E* values. As a consequence, an application of this method would be useful for ranking tentative structures in large supramolecular arrangements [53].

## 4. Materials and Methods

All involved monomers and dimers were considered in their neutral singlet ground states.

Initial structures of stacked homodimers of (4-(azulen-6-yl)phenyl)(methyl)sulfane (denoted as “AZ1” in the preceding section), methyl(4-(2-(methylthio)azulen-6-yl)phenyl)sulfane (“AZ2”), (3-(azulen-6-yl)phenyl)(methyl)sulfane (“AZ3”), and methyl(4-(naphthalen-2-yl)phenyl)sulfane (“NA1”) were prepared using the interactive computer graphics [54]. They were subjected to B97-D/def2-TZVPP [47,48,49] energy optimization and, if converged, to the harmonic vibrational analysis at this level while using default algorithms and settings of the Gaussian 16, revision C.01 suite of codes [55]. This Gaussian software was also employed to obtain the counterpoise-corrected [56] (CP) Δ*E* data by the DC DFT methods, namely, the B3LYP-D3 approach (the standard B3LYP [57,58,59] combination of functionals applied together with the unmodified D3 empirical dispersion-correction term [60]) method combined with the def2-QZVPPD basis set from reference [61] (B3LYP-D3/def2-QZVPPD); by the B2PLYP-D3(BJ) approach (the double-hybrid B2-PLYP functional [33,62] applied together with the Becke–Johnson damping [63] of the D3 term) method combined with the def2-QZVPPD basis set (B2PLYP-D3/def2-QZVPPD); and by the B97-D/def2-TZVPP method referenced above. Data for the Δ*E* estimation by the composite ωB97X-3c/vDZP strategy [34] were obtained using the ORCA 5.0.3 program package [64]. Input was prepared by the “o4wb3c.f” code downloaded from GitHub [65].

In the following, abbreviations “aTZ”, “aQZ”, and “a5Z” denote the standard augmented correlation-consistent polarized valence triple-zeta, quadruple-zeta, and quintuple-zeta basis sets, respectively [40,41]. The CP canonical CCSD(T)/CBS interaction energy was obtained by applying the focal-point method expressed by Equation (1):

(1) $$∆E={∆E}_{HF}^{a5Z}+{∆E}_{MP2\;corr.}^{a5Z}+{∆E}_{post-MP2}^{aTZ}.$$

In this equation, subscripts denote the respective energy terms, namely, the total Hartree–Fock energy (abbreviated as HF), the MP2 correlation energy (“MP2 corr.”), and the higher-order correlation energy (“post-MP2”, often called “δ term” [66], which is taken as a difference of the CCSD(T) and MP2 contributions to the total energy), and superscripts specify the basis set used to compute the respective term (see Section 2.6 of the review [50] for a discussion). The MP2/a5Z correlation energies were obtained in the resolution-of-the-identity integral approximation [67,68] that was applied together with pertinent auxiliary basis sets [68]. Calculations of the HF/a5Z and MP2/a5Z energies were carried out in Turbomole, version 7.1 [69], while the canonical CCSD(T)/aTZ and MP2/aTZ correlation energies were computed in Molpro 2021.2 [70].

The CP DLPNO-CCSD(T)/CBS interaction energy was estimated using the focal-point procedure (see reference [43]) described by Equation (2), where the notation is as in Equation (1), and the right arrow indicates an application of the two-point extrapolation formula from reference [71]:

(2) $$∆E={∆E}_{HF}^{aQZ}+{∆E}_{MP2\;corr.}^{aTZ\to aQZ}+{∆E}_{post-MP2}^{aTZ\to aQZ}.$$

In this case, however, the underlying CCSD(T) and MP2 correlation energies were obtained in the DLPNO approximation [29,30,31,32,38]. The ORCA 5.0.3 program package was used with the default method of the orbital localization and with “T1” option for the iterative treatment of triple excitations within the CCSD(T) method (see reference [32]), while the electron-correlation space was truncated through the “TightPNO” set of parameters.

The density-fitting variant [72] of SAPT-DFT/CBS computations was used as implemented in Molpro 2021.2 and described in detail in our previous work [42]. This approach partitions the total intermolecular interaction energy, *E*, according to Equation (3):

(3) $$E=E_{elst}+E_{exch}+E_{disp}+E_{ind},$$

where $E_{elst}\;$and $E_{exch}$ are the polarization and exchange energy contributions, respectively, arising in the first order of the SAPT expansion [73]; $E_{disp}$ is the dispersion energy contribution obtained as a sum of the second-order dispersion and the second-order exchange–dispersion terms [74]; and $E_{ind}$ is the induction energy contribution taken as a sum of the second-order induction and the second-order exchange–induction terms [75] and of an estimate of all higher-order contributions, which is computed at the HF level [76] (see also reference [77]). These $E_{disp}$ and $E_{elst}\;$data were used to obtain a $\left(E_{disp}/E_{elst}\right)$ ratio.

## 5. Conclusions

A multifarious testing set is proposed, which is termed Set50-50, that consists of dimers ranging from 4 to 50 atoms in size and with values of the dispersion-to-polarization ratio (as estimated by the SAPT-DFT/CBS calculations) between ca. 0.3 and 4.7. The “golden standard” CCSD(T)/CBS Δ*E* values, whose computations involved the canonical CCSD(T)/aug-cc-pVTZ step, are presented for the Set50-50 together with the results of the focal-point DLPNO-CCSD(T)/CBS calculations, two DC DFT methods applied together with a large QZVPPD basis set, and the computationally very cheap ωB97X-3c/vDZP approach. The three DFT-based methods perform comparably for the Set50-50. This obviously favors an application of the computationally cheapest ωB97X-3c/vDZP method to screening of the PES of supramolecular junctions. The DLPNO-CCSD(T)/CBS data are shown to be fully reliable and can be expected to provide an accuracy of better than two kJ/mol for absolute values of Δ*E* in general.

## Figures and Tables

**Table 1:** Computational results for dimers containing up to 32 atoms. The type of intermolecular binding is denoted as “E”, “M” and “D” for electrostatics-dominated, mixed and van der Waals dispersion-dominated complexes, respectively.

Dimer (Configuration)	Type $\left(E_{disp}/E_{elst}\right)$	Negative of the Predicted Δ*E*/kJ/mol				
		CCSD(T)/CBS	DLPNO-CCSD(T)/CBS	B3LYP-D3/def2-QZVPPD	B2PLYP-D3(BJ)/def2-QZVPPD	ωB97X-3c/vDZP
HCl:water	E (0.346)	23.532	23.200	24.405	23.613	23.661
HCN:HF	E (0.278)	31.170	31.308	32.789	32.455	31.898
NCH:FH	E (0.337)	12.256	12.309	13.203	12.338	12.611
HCN:HCN	E (0.297)	19.501	19.578	19.893	20.122	21.074
NH_3_:NH_3_	E (0.423)	13.287	13.184	13.605	12.899	13.830
1-naphtol:water	E (0.360)	29.864	29.883	30.572	29.494	31.249
1-naphtol:NH_3_	E (0.325)	40.520	40.642	42.330	40.810	43.430
HCl:HCl	M (0.809)	7.940	8.148	8.824	8.480	7.154
ethyne:ethyne (T-shaped)	M (0.762)	6.262	6.257	6.920	6.350	6.651
benzene:water	M (1.090)	13.435	13.245	14.361	12.774	13.754
anisole:CO_2_	M (0.918)	15.860	15.551	15.807	14.135	15.662
anisole:NH_3_	M (0.975)	12.003	11.829	12.750	11.053	12.189
pyridine: pyridine (CH…N)	M (0.827)	17.464 (17.431) ^1^ (17.544) ^2^	17.490	16.728	16.565	18.067
1-naphtol:ethyne	M (0.693)	16.964	16.886	17.268	16.803	17.420
uracil:uracil (stacked)	M (1.272)	40.570 (40.246) ^1^ (40.652) ^2^	39.204	42.325	39.980	43.598
aniline:CH_4_	D (2.625)	6.841	6.606	7.159	6.074	7.336
anisole:CH_4_	D (2.615)	7.387	7.118	7.642	6.463	8.308
1-naphtol:CO	D (2.159)	8.374	8.150	8.929	9.228	9.406
1-naphtol:CO_2_	D (1.884)	12.678	12.507	12.010	12.116	13.223
pyridine: pyridine (T-shaped)	D (1.702)	14.521 (14.393) ^1^ (14.548) ^2^	14.603	14.900	14.214	15.763
pyridine: pyridine (stacked)	D (2.225)	15.462 (15.104) ^1^ (15.732) ^2^	14.818	14.404	16.292	16.983
1-naphtol:CH_4_	D (2.604)	9.138	8.858	9.167	8.924	10.715
anisole:anisole	D (1.759)	27.160	26.663	26.492	25.563	26.490

^1^ The “sterling silver” level result from reference [23]. ^2^ The “14k-GOLD” level result from reference [26].

**Table 2:** Comparison of maximum absolute differences and (in parentheses) standard deviations of computational results taken relative to the benchmark interaction energy values, which are specified in the text, for dimers containing up to 32 atoms. All values are in kJ/mol. The type of intermolecular binding is denoted as “E”, “M” and “D” for electrostatics-dominated, mixed and van der Waals dispersion-dominated complexes, respectively.

Type of Dimers	Method			
	DLPNO-CCSD(T)/CBS	B3LYP-D3/def2-QZVPPD	B2PLYP-D3(BJ)/def2-QZVPPD	ωB97X-3c/vDZP
E	0.165 (0.333)	0.572 (1.810)	0.585 (1.285)	0.960 (2.910)
M	0.483 (1.366)	0.740 (1.755)	0.699 (1.725)	1.114 (3.028)
D	0.216 (0.645)	0.602 (1.058)	0.842 (1.597)	0.727 (1.577)
all	0.334 (1.366)	0.759 (1.810)	0.763 (1.725)	0.935 (3.028)

**Table 3:** Computational results for dimers composed of 9*H*-xanthene and either phenol or toluene.

The Second Component	Configuration Number ^1^ $(E_{disp}/E_{elst}$)	Negative of the Predicted Δ*E*/kJ/mol				
		CCSD(T)/CBS	DLPNO-CCSD(T)/CBS	B3LYP-D3/def2-QZVPPD	B2PLYP-D3(BJ)/def2-QZVPPD	ωB97X-3c/vDZP
phenol	1 (2.130)	34.255	34.116	33.820	35.000	33.437
	2 (2.353)	32.675	32.400	33.042	33.813	32.334
	3 (2.716)	30.297	29.609	29.651	32.378	29.346
	4 (3.022)	29.490	28.755	29.202	31.328	28.228
	5 (2.712)	30.165	29.577	29.583	32.145	28.640
	6 (2.170)	31.082	30.847	31.134	32.378	30.430
	7 (2.604)	30.011	29.607	30.243	32.052	29.353
	8 (2.243)	28.723	29.072	28.041	29.800	28.760
toluene	1 (1.990)	40.409	39.326	40.725	42.072	39.239
	2 (1.753)	41.809	40.974	42.160	42.907	40.688
	3 (2.490)	36.918	36.127	36.066	38.767	36.252
	4 (1.826)	41.524	40.965	41.535	42.002	40.589
	5 (2.339)	37.057	36.159	37.396	39.216	35.400
	6 (2.660)	34.524	33.512	33.756	36.437	33.696
	7 (2.814)	33.811	33.152	33.571	35.475	32.745
	8 (2.755)	32.919	32.363	32.095	34.815	31.712
	9 (2.771)	33.081	32.438	32.200	34.821	32.545
	10 (2.235)	34.021	33.697	33.549	34.681	33.120
	11 (1.940)	34.470	34.429	34.684	35.013	34.288

^1^ Numbered consecutively as in reference [46].

**Table 4:** Results of the linear regression of the predicted interaction energy data for eight configurations of 9*H*-xanthene⋯phenol and eleven configurations of 9*H*-xanthene⋯toluene. The model is $\left\{y\right\}=a\times \left\{x\right\}+b$, where $\left\{x\right\}$ is shorthand notation for the canonical CCSD(T)/CBS data, and symbols a, b, σ and $\left|r_{max}\right|$ stand for a slope, intercept, standard deviation of residuals and an absolute value of the maximum residual of this model, respectively.

$\mathrm{Method}\;\mathrm{to}\;\mathrm{Obtain}\;\left\{y\right\}$	Statistical Parameter				
	a	b/kJ/mol	σ/kJ/mol	$\left|r_{max}\right|$/kJ/mol	$\mathrm{Adjusted}\;R^{2}$
DLPNO-CCSD(T)/CBS	0.9952	–0.994	0.315	0.643	0.9927
B3LYP-D3/def2-QZVPPD	1.0461	1.822	0.429	0.731	0.9888
B2PLYP-D3(BJ)/def2-QZVPPD	0.9567	–2.941	0.536	0.905	0.9792
ωB97X-3c/vDZP	0.9714	–0.118	0.416	0.772	0.9878

**Table 5:** Computational results for dimers containing more than 40 atoms. The abbreviations “S-T1” and “PY-2” are used for 2-(4-(methylthio)phenyl)thiophene and 4-(4-(methylthio)phenyl)pyridine, respectively. The type of intermolecular binding is denoted as “D” and “M” for van der Waals dispersion-dominated and mixed electrostatics-dispersion complexes, respectively.

Dimer (Configuration)	Type $\left(E_{disp}/E_{elst}\right)$	Negative of the Predicted Δ*E*/kJ/mol				
		CCSD(T)/CBS	DLPNO-CCSD(T)/CBS	B3LYP-D3/def2-QZVPPD	B2PLYP-D3(BJ)/def2-QZVPPD	ωB97X-3c/vDZP
*n*-heptane: *n*-heptane	D (4.710)	22.525	21.729	25.53	20.588	18.6710
S-T1:S-T1 (#1)	D (2.652)	46.455	46.133	48.051	50.956	42.6430
S-T1:S-T1 (#2)	D (2.458)	47.180	47.014	48.462	51.858	43.3620
S-T1:S-T1 (#3)	D (2.249)	46.297	46.118	47.147	51.123	42.2000
S-T1:S-T1 (#4)	D (2.021)	43.604	43.310	43.805	48.467	39.0240
S-T1:S-T1 (#5)	D (1.791)	38.868	38.880	37.800	43.923	33.4100
PY-2:PY-2 (stacked)	D (1.740)	52.099	52.385	54.432	55.783	51.6390
PY-2:PY-2 (H-bonded)	M (0.961)	18.302	18.257	16.925	16.950	18.7810

**Table 6:** Results of the linear regression of the predicted interaction energy data for eight largest dimers from the Set50-50. The model is $\left\{y\right\}=a\times \left\{x\right\}+b$, where $\left\{x\right\}$ is shorthand notation for the canonical CCSD(T)/CBS data, and symbols a, b, σ and $\left|r_{max}\right|$ stand for a slope, intercept, standard deviation of residuals and an absolute value of the maximum residual of this model, respectively.

$\mathrm{Method}\;\mathrm{to}\;\mathrm{Obtain}\;\left\{y\right\}$	Statistical Parameter				
	*a*	*b*/kJ/mol	*σ*/kJ/mol	$\left|r_{max}\right|$/kJ/mol	$\mathrm{Adjusted}\;R^{2}$
DLPNO-CCSD(T)/CBS	1.0113	0.632	0.280	0.419	0.9994
B3LYP-D3/def2-QZVPPD	1.0340	0.487	1.485	2.732	0.9844
B2PLYP-D3(BJ)/def2-QZVPPD	1.2130	5.339	1.291	2.132	0.9914
ωB97X-3c/vDZP	0.9516	1.291	1.983	3.354	0.9677

**Table 7:** Results of the linear regression of the predicted interaction energy data for the Set50-50.

Method	Difference Type			
	Average Absolute/kJ/mol	Maximum Absolute/kJ/mol	Average Relative	Maximum Relative
DLPNO-CCSD(T)/CBS	0.371	1.366	1.5%	4.4%
B3LYP-D3/def2-QZVPPD	0.739	3.011	3.3%	11.8%
B2PLYP-D3(BJ)/def2-QZVPPD	1.471	5.055	5.3%	14.3%
ωB97X-3c/vDZP	1.293	5.459	5.2%	20.6%

## Data Availability

The data presented in this study are available in the article and in the Supplementary Materials.

## Supplementary Materials

The supporting information can be downloaded at: https://www.mdpi.com/article/10.3390/ijms25010602/s1.
