# Supplementary material for: Reliable Dimerization Energies for Modeling of Supramolecular Junctions
Source: Int J Mol Sci. 2024 Jan 2;25(1):602. doi: 10.3390/ijms25010602 (PMC10778993; doi:10.3390/ijms25010602)
Supplement: Supplementary file 1 [file ijms-25-00602-s001.zip › SI.pdf]

Supplementary Information to "Reliable Dimerization Energies for Modeling of Supramolecular Junctions" by Czernek & Brus (submitted to *IJMS* in 2023)

Table of Contents

Table S1 at page S2: Description of the employed geometry of complexes whose size does not exceed 32 atoms

Figure S1 at page S3: The B97-D/def2-TZVPP minima of stacked and H-bonded dimers of "PY-2"

Table S2 at page S4: Computational results for eight dimers that are not a part of the Set50-50

Table S3 at page S4: Results of the linear regression of the predicted interaction energy data for eight dimers that are not a part of the Set50-50

Table S1. Description of the geometry of investigated complexes whose size does not exceed 32 atoms.

**Dimer (Configuration)**	**Geometry**
HCl:water	MP2/aTZ from reference [42] of the main text
HCN:HF	MP2/aTZ from reference [43] of the main text
NCH:FH	MP2/aTZ from reference [43] of the main text
HCN:HCN	MP2/aTZ from reference [43] of the main text
NH_3:NH_3	MP2/aTZ (this work)
1-naphtol:water	MP2/aTZ from reference [43] of the main text
1-naphtol:NH_3	MP2/aTZ from reference [42] of the main text
HCl:HCl	MP2/aTZ from reference [42] of the main text
ethyne:ethyne (T-shaped)	MP2/aTZ from reference [43] of the main text
benzene:water	MP2/aTZ from reference [42] of the main text
anisole:CO_2	MP2/aTZ from reference [43] of the main text
anisole:NH_3	MP2/aTZ from reference [42] of the main text
pyridine:pyridine (CH…N)	MP2/cc-pVTZ downloaded from http://www.begdb.org/
1-naphthol:ethyne	MP2/aTZ from reference [42] of the main text
uracil:uracil (stacked)	MP2/cc-pVTZ downloaded from http://www.begdb.org/
aniline:CH_4	MP2/aTZ from reference [42] of the main text
anisole:CH_4	MP2/aTZ from reference [42] of the main text
1-naphtol:CO	MP2/aTZ from reference [42] of the main text
1-napthol:CO_2	MP2/aTZ from reference [42] of the main text
pyridine:pyridine (T-shaped)	MP2/cc-pVTZ downloaded from http://www.begdb.org/
pyridine:pyridine (stacked)	MP2/cc-pVTZ downloaded from http://www.begdb.org/
1-naphtol:CH_4	MP2/aTZ from reference [42] of the main text
anisole:anisole	MP2/aTZ from reference [42] of the main text

Figure S1. The B97-D/def2-TZVPP minima of stacked and H-bonded dimers of “PY-2” shown together with the length scale automatically generated in the Materials Studio 2019.

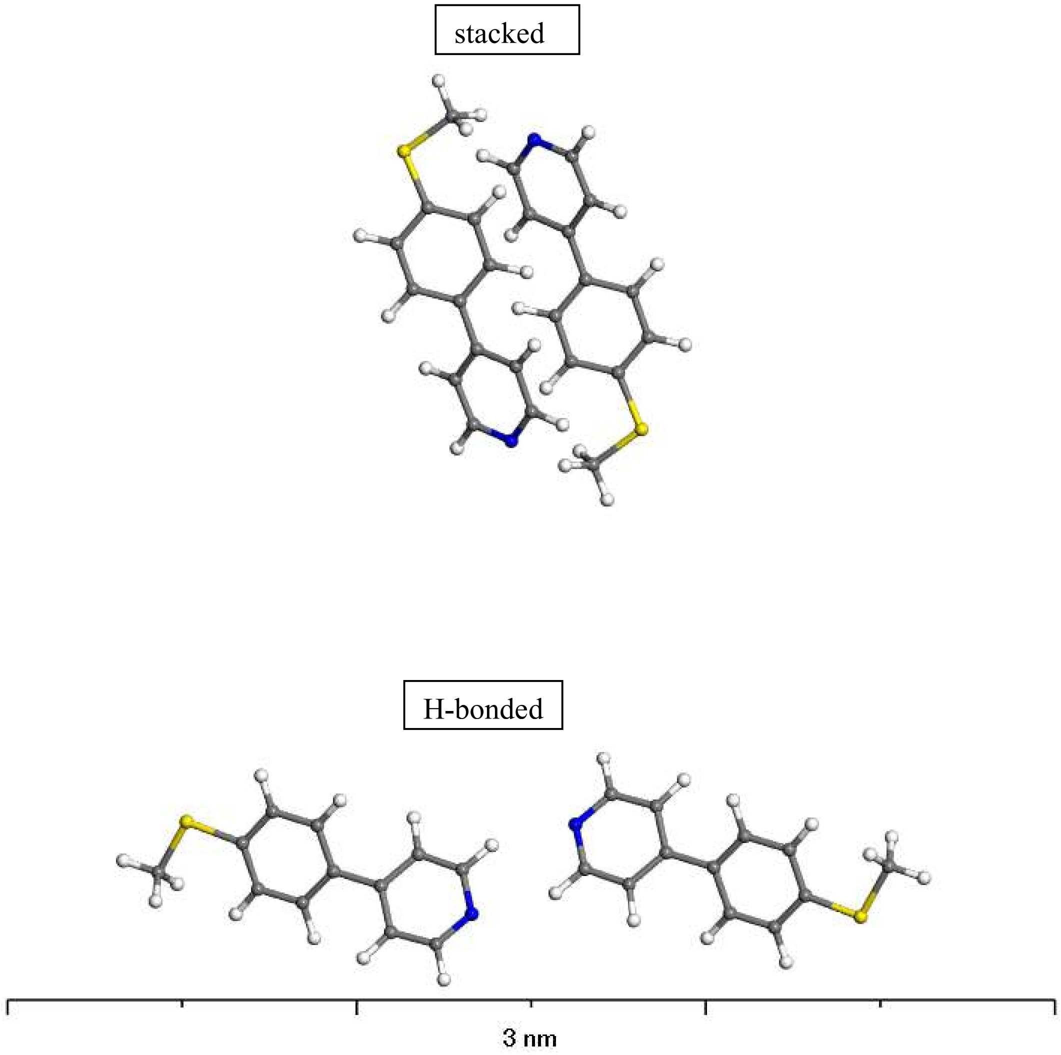

Table S2. Computational results for eight dimers that are not a part of the Set50-50. See the main text for chemical names of "AZ1", "NA1", "AZ2" and "AZ3". The type of intermolecular binding is denoted as "D" to indicate its van der Waals dispersion character.

Dimer (Configuration)	Type ($E_{\mathrm{disp}}/E_{\mathrm{elst}}$)	Negative of the Predicted ΔE/ kJ/mol				
		DLPNO-CCSD(T)/CBS	ωB97X-3c/vDZP	B97-D/def2-TZVPP	B3LYP-D3/def2-QZVPPD	B2PLYP-D3(BJ)/def2-QZVPPD
AZ1:AZ1 (#1)	D (2.610)	67.757	60.218	65.731	65.814	73.304
AZ1:AZ1 (#2)	D (2.337)	67.466	61.969	65.312	66.986	71.421
NA1:NA1 (#1)	D (3.129)	62.341	56.181	61.965	62.760	69.705
NA1:NA1 (#2)	D (3.128)	59.115	53.837	56.066	56.693	65.689
AZ2:AZ2 (#1)	D (2.496)	84.465	72.140	76.316	81.463	90.625
AZ2:AZ2 (#2)	D (2.500)	80.520	69.708	73.889	77.656	86.567
AZ3:AZ3 (#1)	D (2.931)	69.455	62.940	66.024	68.576	75.479
AZ3:AZ3 (#2)	D (2.671)	66.452	60.141	63.890	65.814	70.542

Table S3. Results of the linear regression of the predicted interaction energy data for eight dimers that are not a part of the Set50-50 (see Table S1). The model is $\{y\} = a \times \{x\} + b$, where $\{x\}$ is shorthand notation for the canonical CCSD(T)/CBS data, and symbols a, b, σ and $|r_{\mathrm{max}}|$ stand for a slope, intercept, standard deviation of residuals and an absolute value of the maximal residual of this model, respectively.

Method to Obtain $\{y\}$	Statistical Parameter				
	a	b / kJ/mol	σ / kJ/mol	$|r_{\mathrm{max}}|$ / kJ/mol	adjusted R^2
ωB97X-3c/vDZP	0.7139	–12.386	0.823	1.420	0.97954
B97-D/def2-TZVPP	0.7326	–15.089	1.134	2.231	0.96358
B3LYP-D3/def2-QZVPPD	0.9123	–4.367	1.000	1.874	0.98144
B2PLYP-D3(BJ)/def2-QZVPPD	0.9969	–5.935	1.172	1.772	0.97870
